# Sexual dimorphic effects of *igf1* deficiency on metabolism in zebrafish

**DOI:** 10.3389/fendo.2022.879962

**Published:** 2022-07-29

**Authors:** Ningmei Zeng, Jiankang Bao, TingTing Shu, Chuang Shi, Gang Zhai, Xia Jin, Jiangyan He, Qiyong Lou, Zhan Yin

**Affiliations:** ^1^ State Key Laboratory of Freshwater Ecology and Biotechnology, Institute of Hydrobiology, Chinese Academy of Sciences, Wuhan, China; ^2^ University of Chinese Academy of Sciences, Beijing, China; ^3^ Chinese Sturgeon Research Institute, China Three Gorges Corporation, Yichang, China; ^4^ The Innovative Academy of Seed Design, Chinese Academy of Sciences, Beijing, China

**Keywords:** sexual dimorphism, hepatic metabolism, glucose uptake, lactate/pyruvate ratio, *igf1*

## Abstract

Insulin-like growth factor 1 (IGF1) is an essential effector of the growth hormone (GH)/IGF1 axis for somatic growth regulation in mammals. However, its functions have not been thoroughly investigated in zebrafish *in vivo*. In this study, the *igf1*-deficient zebrafish model was developed using the CRISPR/Cas9 technique. In this study all the results were performed on both male and female animals. The growth of both male and female *igf1*-deficient zebrafish were reduced. The *igf1* deficiency leads to significant complementary up-regulation of transcriptional expression levels of *insulin, igf2* and *igf3*. This suggested that *igf2* and *igf3* may act with redundant functions. While the upregulation of *gh1* expression can only be detected in *igf1*-deficient females. At the same time, significant growth retardation, fatty liver, reduced activated levels of ribosomal S6 (S6) are seen only in *igf1-*deficient males. On the other hand, significant hyperglycemia, elevated transcriptional expression levels of *phosphenolpyruvate carboxykinase* (*pepck*) and levels of phosphorylated extracellular signal-regulated kinase (ERK1/2), with additional reduced hepatic lactate/pyruvate (L/P) ratios can only observed in *igf1*-deficient females. Impaired glucose uptake has been recorded in the primary cultured hepatocytes from igf1-deficient females, but not males. Intriguingly, exposure to 17beta-estroadiol (E2) can partially ameliorated the defects of fatty liver and activation of AKT/mTOR signaling in *igf1*-deficient males. Our studies demonstrate the significant functions of IGF1 on somatic regulation in zebrafish, with asymmetric gender-related consequences. Our data thus suggest that the zebrafish IGF1 is preferentially required for the activation of AKT/mTOR signaling in male zebrafish, but glucose uptake in females.

## Introduction

Insulin-like growth factor 1 (IGF1) is an insulin-like peptide (ILP) (1). In vertebrates, ILP signals can activate the canonical insulin signaling pathway through hybrid dimer receptors with different affinities for insulin and IGFs comprising different forms of receptor tyrosine kinase. The typical insulin/IGFs signaling pathway includes downstream molecules such as insulin receptor substrate (IRS), phosphoinositol 3-kinase (PI3K)/AKT, target of rapamycin (TOR), mitogen-activated protein kinases (MAPKs) including extracellular signal-regulated kinase (ERK) (2, 3).

IGF1 acts as a downstream effector of growth hormone (GH) signaling in verterbrate, thereby forming the somatotropic (GH/IGF1) axis together with GH (4). This is believed to be the major endocrine axis of somatic growth (5). Most studies on mice with complete *igf1*-deficiency have reported poor viability (6, 7), with a few observations of postnatal progressive growth deficits in humans and in mice homozygous for the *igf1* gene mutation (8). To investigate IGF1 functions on postnatal growth, several studies have been conducted with models harboring tissue-specific deletion of IGF1 signaling. In mammals, the endocrine form of IGF1 is mainly produced and secreted by the liver. However, hepatic IGF1 production in deficient mice generated *via* the Cre/loxP system proceeds normally, with sexual dimorphism in a GH-stimulation manner. It has been reported that hepatic IGF1-deficient female mice can respond to GH for postnatal growth, while their male counterparts are resistant to GH stimulation (9). Utilizing an experimental mouse model of IGF receptor deficiency *via* dosage deletion of the floxed *igf1r* gene, sexually dimorphic growth deficits were observed in mice, particularly affecting the male fat tissue (10). Taken together, these observations indicate the sexual dimorphic effects of IGF1 on growth in mammals.

The functions of IGF1 in teleost was considered for the midline and notochord development in the early gastrulation stage (11, 12). Using our *igf1* overexpression in skeletal muscle transgenic crucian carp, it has been demonstrated that teleost IGF1 functions on promotion of the anabolic process, lipid utilization, and aerobic metabolism (13). However, no observation has been made on the effects of teleost *igf1*-deficiency on postnatal growth. The existence of dwarfism without significantly altered *igfs* transcriptional expression, as observed in *gh1* zebrafish mutant *vizzini* at the larval stage, suggests the difference between the modules of the GH/IGF axis in teleost and mammals on postembryonic somatic growth (14).

The endocrine hormones such as estrogen and androgen showed the obviously asymmetric gender-related distribution. It has been suggested that GH action, such as its secretion, can be enhanced by estrogen signaling (15–17). In an analysis of zebrafish and human hepatocyte, estrogen could activate downstream effectors of the PI3K/TOR signaling to promote hepatocyte proliferation through GPER1 (18) which was a G-protein related receptor. In biomedical studies growing number of sex related differences were raised including both gene dosage effect of the X and Y chromosomes and the metabolism. In fish, sexual dimorphic phenotypes in terms of growth performance has been caught increasingly concerns of investigators recently.

To clarify the function of IGF1 on teleost growth regulation, we developed an *igf1*-deficient zebrafish model using the CRISPR/Cas9 technique. With this model, sexually dimorphic patterns of postnatal growth retardation and metabolic alterations has been evidently observed. Our present studies provide evidence indicating the sexually dimorphic functions of IGF1 for the activation of AKT/TOR signaling and glucose uptake in zebrafish.

## Materials and methods

### Zebrafish maintenance and genetic line generation

Zebrafish were maintained in circulating water with a 14-h light and 10-h dark cycle at 28°C. They were fed twice daily with newly hatched brine shrimp. To collect fertilized eggs, mature male and female pairs were transferred into the tanks before the end of the light period. After the beginning of the next light cycle, partitions were moved to allow for laying eggs and fertilization. Embryos were then collected, and stored in water containing 0.006% ocean salt (19). The larval containing wildtype, heterozygous and homozygous were maintained and raised in the same tank. Adult zebrafish at 90 days post-fertilization (dpf) were used for the assays conducted in this study following genotyping. The body weight data were collected using precision balance followed by wiping dry with gauze. The food intake were caculated with the body weight between meals. All animal experiments were conducted in accordance with the Guiding Principles for the Care and Use of Laboratory Animals and were approved by the Institute of Hydrobiology, Chinese Academy of Sciences (Approval ID: IHB 2013724).

### *Igf1* targeting *via* CRISPR/Cas9

The CRISPR/Cas9 technique was used for gene editing in zebrafish according to the method described by Mali (20). Guide RNA was synthesized using the TranscriptAid T7 High Yield Transcription kit (Thermo, K0441) in accordance with the gene sequence information of zebrafish *igf1* in the NCBI database (AC. No. BX510924.11). Cas9 mRNA was synthesized using the mMESSAGE mMEACHINE mRNA transcriptional synthesis kit (Ambion, AM1344). gRNA and mRNA were mixed and then injected into zebrafish fertilized eggs at a 1-2-cell stage. Two independent *igf1* mutant lines were screened *via* DNA sequencing. The phenotypes of these *igf1* mutants were similar; therefore, most of our experiments were performed using the *igf1* mutant line 2 (M2), unless stated otherwise.

### Western blotting and ELISA analysis

Western blot analysis was performed following the methods described previously (21, 22). Briefly, zebrafish hepatic samples were lysed in RIPA buffer containing proteases and phosphatase inhibitors. Total protein in samples were separated by gel SDS-PAGE and transferred onto the Protran Nitrocellulose Transfer membrane (Immobilon^®^-P). The membranes were blocked for 2 h in TBS/0.1% Tween/5% milk. The primary antibodies used were: β-Actin (Santa Cruz, SC-69879), Igf1(Abcam, ab106836), S6 Ribosomal Protein (Cell Signaling, 2217), p-S6 (Cell Signaling, 2215), Akt (Cell Signaling, 9272), p-Akt (Cell Signaling, 4060), Ampk (Cell Signaling, 2532), p-Ampk (Cell Signaling, 2531), Mapk (Cell Signaling, 4695), p-Mapk (Cell Signaling, 4370). Secondary antibodies used were: Peroxidase-conjugated Affinipure Goat Anti-Mouse IgG (H+L) (ProteinTech, SA00001-1), Peroxidase-conjugated Affinipure Goat Anti-Rabbit IgG (H+L) (ProteinTech, SA00001-2), Peroxidase-conjugated Affinipure Donkey Anti-Goat IgG (H+L) (ProteinTech, SA00001-3). The signal was detected using a CCD camera-based imager (ImageQuant LAS 4000 mini, GE). The intensity of the bands was quantitated using the ImageJ software version 1.49V.

To determine the expression levels of IGF1 peptide in the serum, the blood samples were collected following anesthesia with MS222 (J&K, 247097). The blood samples were added heparin as an anticoagulant with the final concentration to 20 units/ml. The samples were centrifuged for 20 minutes at 1000g at 4°C of collection the supernate. IGF1 ELISA Kit of zebrafish (mlbio, ml365400-2) was used for measurement following the manufacturer’s instructions.

### Quantitative real-time PCR

Total RNA was extracted using the RNeasy Mini kit (Transgen, China). MMLV reverse transcriptase (Thermo, USA) was used for reverse transcription. Primers used in the study are listed in **Table 1**. RT-qPCR was conducted using TransStart**^®^** Tip Green qPCR SuperMix (Transgen, China) with the Bio-Rad (CFX96 Touch) software and calculated by the Bio-Rad CFX Manager 3.1 (23). The results are presented according to the method described by Doom (24). Both *β-actin* and *ef1α* gene were used for internal reference in the Semi-Quantitative Real-time PCR and the fold-change of the target gene should be similar.

**Table 1:** The primers used in the study.

Symbol	NCBI accession	Forward primers(5 ′-3 ′)	Reverse primers(5 ′-3′)
genotyping			
*igf1*	NC.007115.7	CAGCGTGTGATTGTAATGTG	GCACTTACTGAAATAAAAGC
RT-qPCR			
*Igf1*	NM_131825.2	ACTGGTGCTGTGCGTCCTC	GGTCCATATCCTGTCGGTTT
*Igf2a*	NC_007118.7	TCCTTAACCTCTGAGCAGCTTTT	GCAGGTCTTCCCAGTGTCA
*Igf2b*	NC_007136.7	TGTTGTATCATCGGTCTGGG	GTTCATTCTTTGTGGCATCG
*Igf3*	NM_001115050.1	GCCAAACGCCTTCAGATAATGC	GCTGCTCCAGGTTTGCCTATGT
*pepck*	NM_214751.1 106 87 GGTCAACAACTGGCCCTGTA CAGCAGTGAGTTTCCTCCGT	GGTCAACAACTGGCCCTGTA	CAGCAGTGAGTTTCCTCCGT
*gh*	NM_001020492.2	GCATCAGCGTGCTCATCAAG	TGAGACTGGTCTCCCCTACG
*insulin*	NM_131056.1	GGTCGTGTCCAGTGTAAGCA	GGAAGGAAACCCAGAAGGGG
*igf1ra*	NM_152968.1	TTCTCCTGTTCCGTGTCTCC	CCGAATCCAAGTAGCACAGC
*Igf1rb*	NM_152969.1	AGCCTTCGAGAACTTCCTCC	AAACGGTAAAAGGCTGCAGG
*Igfbp1a*	NM_173283.4	CCACCAGTTTCTTGCGTATC	AGCCTAACCACAGCCAAAGCGAG
*Igfbp1b*	NM_001098257.2	ACCACCCTACTGAAGAGGACACAGA	GCGTTGAGTTGTGACTTGATGCTC
*Igfbp3*	NM_205751.2	TGTCGTCGGGAGATGGAAAG	GAAGCGTCTTGGGTTCAGCA
*Igfbp7*	NM_212924.2	GACGGACGGAACTACAACAGC	TGCACCGCCAGATTATCTTTATCTC
Reference gene			
*β-actin*	NM_131031.1	ACTCAGGATGCGGAAACTGG	AGGGCAAAGTGGTAAACGCT
*ef1α*	NM_131263	TACCCTCCTCTTGGTCGCTTT	ACCTTTGGAACGGTGTGATTG

### Oil red o staining

The hepatic tissue was sampled and placed in fixative fluid (Servicebio, G1119). Then, samples were frozen, sectioned, and stained with oil red to detect fat deposits in the hepatic samples (25). The images were acquired using a stereomicroscope (Zeiss SteREO Discovery. V20, Germany).

### 17beta-estroadiol administration and triglyceride

The igf1 deficient zebrafish and wild type control zebrafish were exposed in 1mM 17beta-estroadiol (E2) dissolved in zebrafish egg water for 14 days. The liver tissue was collected for total RNA extraction and TG assay. Tissue samples from zebrafish were homogenized in PBS (0.1 mol/L pH 7.4), and then triglycerides were detected using the Triglyceride Assay Kit following the manufacturer’s instructions (Njjcbio, A110-1-1).

### Body fat content assay (choloform and methanol extraction)

The contents of fish fat were assayed as per previously described procedures (26). The adult zebrafish were anesthetized and frozen at -80°C for 24 h, then dried with a freeze-dryer (Alpha 2-4 LSC). After weighing the dry weight, 6 mL of a mixture of chloroform and methanol (volume 2:1) was added to the samples. Then, the samples were ground and sealed overnight at 4°C to allow the fat to separate fully from the tissue. Afterwards, 2 mL of KCl (0.37 M) was added. The samples were then oscillated for 5 min. After the samples achieved stratification, they were centrifuged at 500g for 20 min. Finally, the liquid in the lower layer was transferred using a Pasteur Pipette into a newly weighed tube. The extraction was first air-dried under nitrogen, and then oven-dried at 55°C. The sample left was the total fat to weigh. Ratios of weight of total fat/dry weight were calculated as the body fat content.

### Measurement of blood glucose

Blood samples for blood glucose measurement were collected an hour after each meal (27). The OneTouch UltraVue (LifeScan) blood-glucose tester was used for the measurement of blood glucose levels, according to the manufacturer’s instructions. The mouse IGF1 recombinant proteinwas purchased from *Cloud-Clone Corp*. The protein was diluted with saline to 50µg/ml/kg.

### Primary cell culture

The hepatic zebrafish samples were collected and placed into Ca^2+^/Mg^2+^-free HBSS Medium (Gibco) for rinsing three times. After the samples were chopped, they were digested in collagenase (VETEC, Type II) at 28°C for 30 min. The cell suspensions were filtered through 0.45-μm filters. Cells were then collected by centrifugation. Counted cells were seeded into plates with Polyethyleneimine (ALDRICH), and cultivated in DMEM/F-12(HAM)1:1 (BI, Biological Industries) with 10% penicillin-streptomycin solution (Biosharp) for 24 h at 28°C in humidified atmosphere with 5% CO2. After 24 h of incubation, cells were used for the glucose uptake assay (28).

### Glucose uptake assay

Primary cultured cells with different treatments were collected and washed twice with PBS. Cells (1× 10^4^) were placed into each well of a 96-well plate, and then processed with reagents for the Glucose Uptake-Glo™ Assay (Promega, J1341) per the manufacturer’s instructions. Luminescence was recorded using 1-s integration on a Dual-Luciferase reporter assay system (Promega), according to the manufacturer’s instructions to measure the qualify the 2DG (2-Deoxy-D-glucose) in the hepatocyte.

### Detection of lactate and pyruvate

The levels of lactate in zebrafish hepatic tissues were determined using the Lactate-Glo™ Assay (Promega, J5021). Luciferase activity was measured using the Dual-Luciferase reporter assay system (Promega). We used the Pyruvate Assay Kit (BestBio, BB47421) to detect pyruvate in hepatic tissues and the results were obtained using the MD-SpectraMax M5. All procedures were performed according to the manufacturer’s instructions.

### Statistical analysis

All data for RT-qPCR and western blotting intensity analyzed by the Image J software were expressed as the mean ± SD. The Student’s *t*-test was used for determining the statistical significance, which was defined as *, *P* < 0.05; **, *P* < 0.01; ***, *P* < 0.001. Statistic calculations were performed using the GraphPad Prism Software. Each result represents the mean of at least three independent experiments.

## Results

### Generation of *igf1-*deficient zebrafish lines

**Figure 1:**
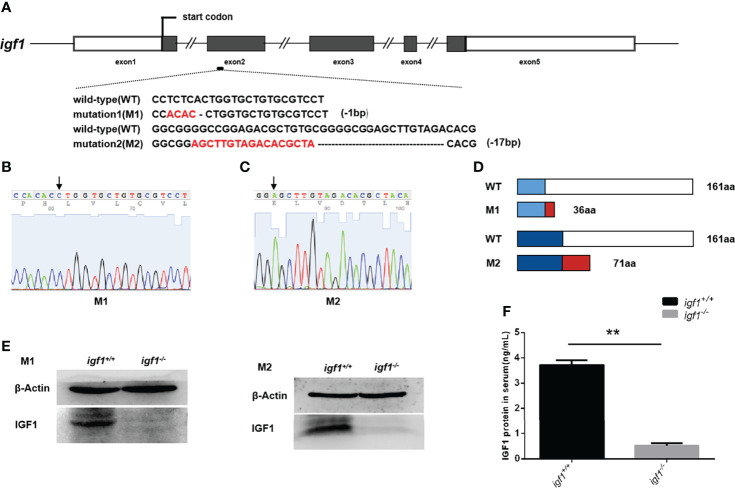
Generation of *igf1-*deficient zebrafish. **(A)** The diagram shows the endogenous structure of the zebrafish *igf1* gene locus and selected targeting region. The dark boxes represent the coding regions the hollow boxes indicate non-translated regions, and the lines represent introns. The target site of CRISPR/Cas9 was located at exon 2. Two mutant lines were obtained at different target sites, namely mutant 1 (M1, -1bp) and mutant 2 (M2, -17bp). **(B, C)** Sequencing image of *igf1* in M1 **(B)** and M2 **(C)**. The arrowhead indicates the upstream border of the deletions. **(D)** Diagram showing the putative peptides of the wild-type IGF1, M1, and M2 peptides. The mutant lines with putative peptides identical to those of WT fish are shown as blue areas, and the red areas indicate miscoded amino acids. **(E)** Western blotting of IGF1 protein in the hepatic organ of mutants and control siblings. Three biological repeats were carried out. **(F)** Circulating IGF1 levels of IGF1 were determined through ELISA. Four animals were sampled for each experiment and three biological repeats were carried out and statistical analysis was performed using a t test (P<0.01, n=3). **P < 0.01.

To investigate the physiological role of *igf1* in zebrafish, functional *igf1*-deficient zebrafish were generated using the CRISPR/Cas9 technique (**Figure 1A**). According to the *igf1* gene sequence information for zebrafish in the NCBI database (AC. No. BX510924.11), we selected a fragment on the second exon of the *igf1* gene as target sites for gene editing (**Figure 1A**). Two independent *igf1* mutant lines, mutant line 1 (M1) and mutant line 2 (M2), were obtained. These mutant lines were caused by a 1-bp and 17-bp deletion within the *igf1* coding region resulting in reading frameshifts and premature stop codon for M1 and M2, respectively (**Figures 1B, C**). The two putative truncated mutant peptides with 36 and 71 amino acids (aa) of M1 and M2 contained substitutions of 27 and 44, non-homologous aa peptides occurring at aa residue 9 and 27, respectively (**Figure 1D**). The diminished IGF1 protein in the hepatic extracts from our two independent *igf1* mutant fish lines were confirmed by western blotting analysis (**Figure 1E**). Since the IGF1 peptide was commonly secreted to the circulation, the levels in plasma were determined using ELISA. The IGF1 protein in the plasma were disrupted compared with wildtype control (**Figure 1F**).

### The feeding back regulation of *gh* (*growth hormone*) and redundant functions of *Insulin*, *igf2*, *igf3* in *igf1* deficient zebrafish

**Figure 2:**
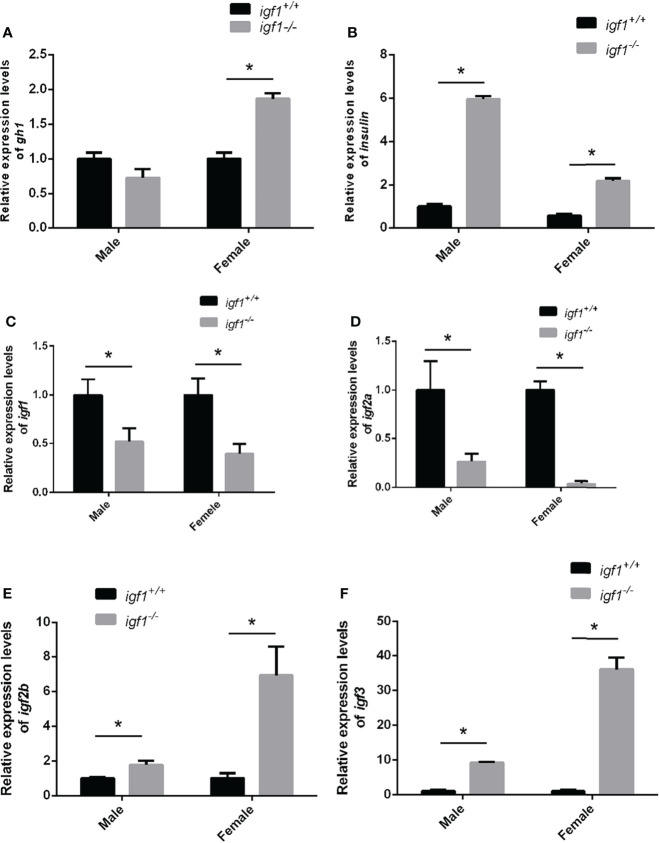
Feeding-back transcriptional levels of igf1 gene **(A, B)** Relative expression levels of *gh1* **(A)** and *insulin* **(B)** in *igf1*-deficient fish and the control wild-type fish and three biological repeats were carried out and statistical analysis was performed using a t test (n=3). **(C–F)** Relative expression levels of *igf1*, *igf2a*, *igf2b* and *igf3* in igf1-deficient fish and the control wild-type fish and three biological repeats were carried out and statistical analysis was performed using a t test (n=3). There was no difference between the two internal reference gene (β-actin and EF1α). Four animals were sampled for each experiment and three biological repeats were carried out and statistical analysis was performed using a t test. The asterisk "*" represented significant difference, P<0.05.

**Figure 3:**
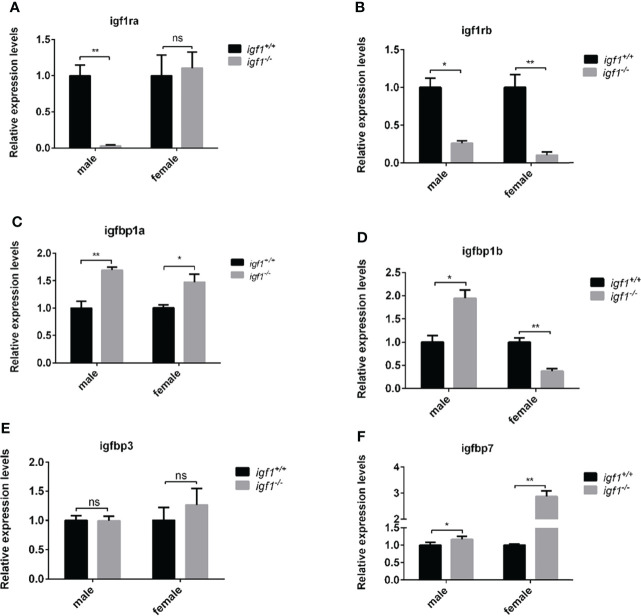
Transcriptional levels of igf1 related genes **(A, B)** Relative expression levels of *igf1ra and igf1rb* in *igf1*-deficient fish and the control wild-type fish and three biological repeats were carried out and statistical analysis was performed using a t test (n=3). **(C–F)** Relative expression levels of *igfbp1a*, *igfbp1b*, *igfbp3* and *igfbp7* in *igf1*-deficient fish and the control wild-type fish and three biological repeats were carried out and statistical analysis was performed using a t test (n=3). There was no difference between the two internal reference gene (β-actin and EF1α). Four animals were sampled for each experiment and three biological repeats were carried out and statistical analysis was performed using a t test (p<0.001, n=3). **P < 0.01.

Considering the crosstalk between GH, IGF1, and Insulin signaling on the fish growth and metabolism of organisms (29, 30), the transcriptional expression of *gh1* in the pituitary gland and *insulin* in the hepatopancreas tissue samples were measured by quantitative Real-time PCR (RT-qPCR). The expression levels of *gh1* remained unchanged in *igf1*-deficient males, while in the mutant females, the expression levels of *gh1* in the pituitary samples were significantly upregulated compared with those of their wild-type siblings (**Figure 2A**). The expression of *insulin* in the liver was significantly upregulated in both males and females *igf1* mutants (**Figure 2B**). At the same time,the expression of the *igf1* homolog genes including igf2 and igf3 were checked using realtime PCR in liver tissue. The result showed that the expression of igf2a/b and igf3 were elevated in both male and female *igf1*-deficient zebrafish (**Figures 2C–F**). At the same time, the expression of igf1 receptor a/b were checked for conprehension of the signaling of *igf1*. The expression of igf1ra was decreased in male *igf1*-deficient zebrafish. While the expression of igf1rb was decreased in both male and female igf1-deficient zebrafish (**Figures 3A, B**). igfbp1, igfbp3 and igfbp7 were considered to be related with glucose and lipid metabolism. We also checked the expression levels of the igfbps. The data showed that the expression of *igfbp1a* and *igfbp7* were increased in both male and female *igf1*-deficient zebrafish. The expression of *igfbp1b* was increased in male *igf1*-deficient zebrafish while decreased in female. The expression of igfbp3 was not influenced (**Figures 3C–F**).

### Sexual dimorphic effects on the growth and metabolism of *igf1*-deficient zebrafish

**Figure 4:**
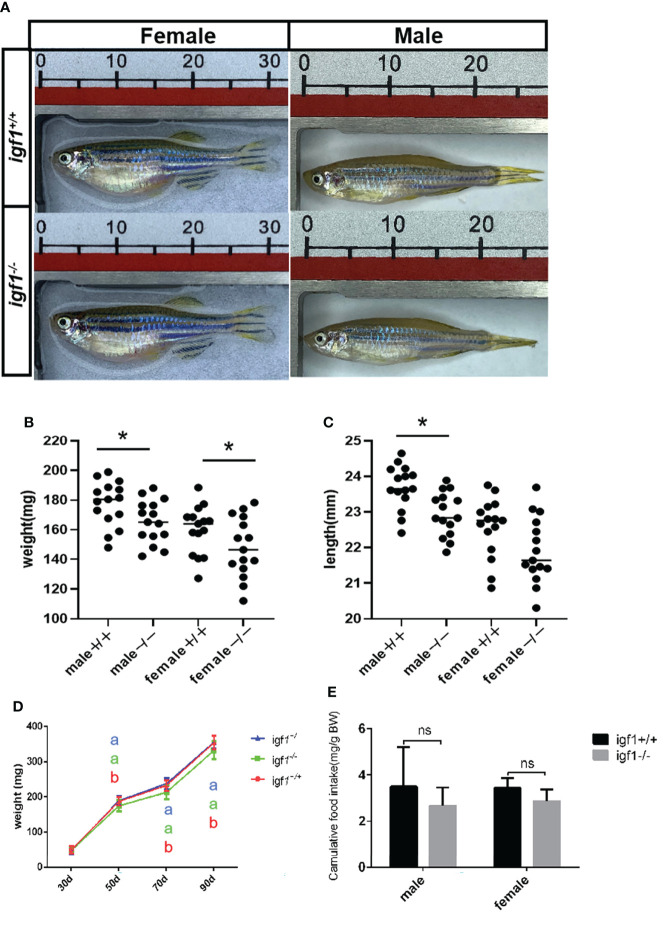
General growth performance traits of *igf1*-deficient fish. **(A)** Appearance of *igf1*-deficient fish and their control wild-type fish at 90 dpf (n=15/group). **(B, C)** Body weight of the fish at the 90-dpf stage. Three times of independent growth analysis were carried out and showed similar results. *P < 0.05. **(D)** Growth curves of wild type, heterozygous and *igf1*-deficient zebrafish during the growth statge. **(E)** The food intake data was showed in the chart. 10 animals were weighted for each experiment and three biological repeats were carried out and statistical analysis was performed using a t test (ns, p>0.05, n=3). The letters a and b in the charts represent significant differences between which labeled with different letters.

The zebrafish for growth comparison data were derived from same F1 heterozygous mating group. Sequencing of the amplified products of the targeting region of the *igf1* locus from the caudal fin genome DNA (gDNA) samples were used for genotyping. The *igf1*-deficient fish and their wild-type control siblings were raised in the same tank for somatic growth observation. The body weight and the body length of the wild-type and *igf1*-deficient fish, including males and females, were examined regularly during the process of growth (**Figures 4A–D**). Both parameters of *igf1*-deficient males was significantly reduced compared to those of the control siblings. There was a similar observation in the *igf1*-deficient females compared to their female control siblings, but the difference was not as drastic as their male counterparts. There was no difference in growth of *igf1* heterozygous mutant could be examined. The food intake of the igf1 *igf1*-deficient zebrafish were examined. There were moderate decrease however with no statistic significant difference in male and female zebrafish (**Figure 4E**).

### Sexual dimorphic effects on hepatic fat accumulation in *igf1*-deficient zebrafish

**Figure 5:**
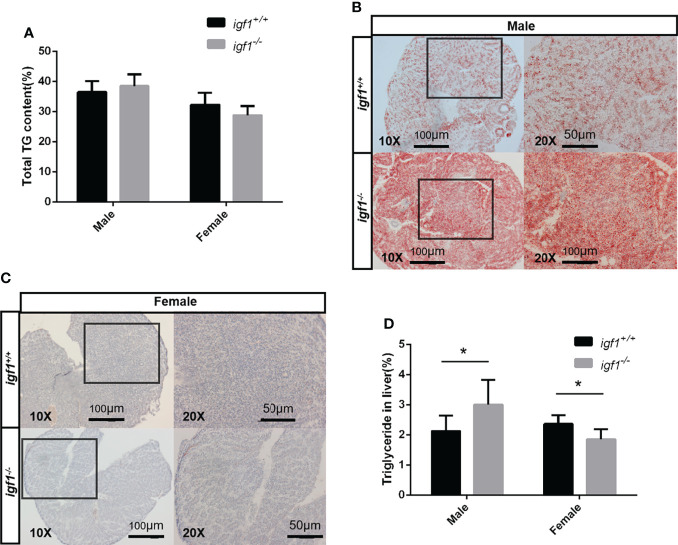
Hepatic steatosis observed in *igf1*-deficient male fish. **(A)** Body fat composition of *igf1*-deficient and wildtype control. **(B, C)** Frozen section and oil-red O staining of the hepatic tissue. Lower magnification images are shown at 10X, higher magnification of the indicated regions is shown at 20X. **(D)** Triglyceride content of the hepatic organ of *igf1*-deficient fish compared to controls (n = 6/group). *P < 0.05.

No observable differences in whole body fat content were recorded between *igf1* mutants and their wild-type controls (**Figure 5A**), based on the chloroform and methanol extration and micro-CT methods (data not shown). To further assess the possible association between *igf1* deficiency and metabolic disorders (31), features of hepatic tissues were examined. We observed that the hepatocytes of *igf1*-deficient males contained more triglyceride (TG), while the mutant females showed no differences, compared to their control counterparts, as analyzed *via* oil red O staining of the hepatic tissue sections (**Figures 5B, D**). This was subsequently verified by assessing the TG content in hepatic tissue, which was significantly up-regulated in *igf1*-deficient males, but not in females (**Figures 5C, D**).

### Sexual dimorphic effects on glucose metabolism of *igf1*-deficient zebrafish

**Figure 6:**
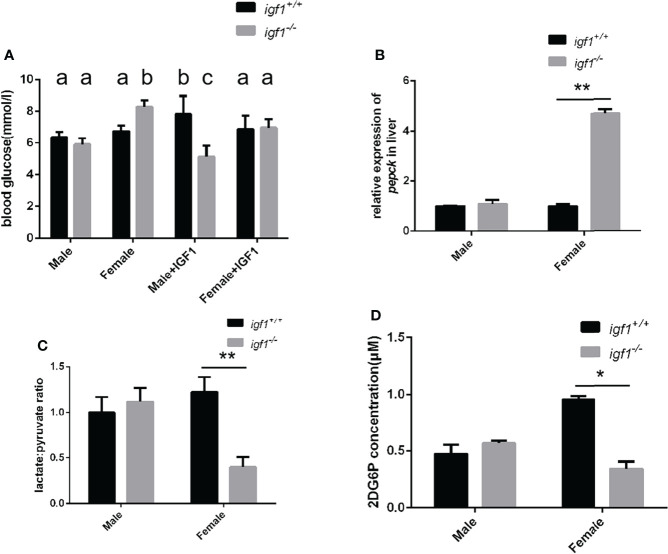
Alterations of glucose metabolism in *igf1*-deficient fish. **(A)** Postprandial plasma glucose levels (1 h after each meal) of *igf1*-deficient fish and sdminastration of recombinanat IGF1 protein(n = 6/group). The letters a, b and in the charts represent significant differences between which labeled with different letters. **(B)** Relative expression of *pepck* gene. **(C)** The hepatic lactate/pyruvate ratios of *igf1*-deficient fish (n = 8/group). **(D)** The hepatic 2DG6P levels of *igf1*-deficient fish. *P < 0.05; **P < 0.01.

We analyzed the blood glucose levels of zebrafish using a blood glucose detector 1 h after each meal. The blood glucose levels of *igf1*-deficient males were significantly decreased while those of *igf1*-deficient females were significantly increased compared to their respective control groups (**Figure 6A**). The hyperglycemia phenotype of female *igf1*-deficient could be rescued with recombinant IGF1 protein through intraperitoneal injection. The blood glucose of male igf1-deficient zebrafish was not affected and furthermore administration of IGF1 protein caused decreased blood glucose level (**Figure 6A**). The transcriptional level of key enzyme coding gene in gluconeogenesis *pepck* was checked using realtime PCR (**Figure 6B**). Slightly increased hepatic L/P ratios were observed in male *igf1*-deficient fish compared to control fish. In contrast, significantly reduced hepatic L/P ratios were observed in *igf1*-deficient females compared to their female control siblings (**Figure 6C**). Primary cultured hepatocytes were used to test their glucose absorption capacity. No significant alteration in cellular 2DG6P content which revealed the hepatic glucose uptake was observed in male hepatocytes compared to wild-type control fish. However, there was significantly decreased glucose absorption capacity in the mutant female hepatocyte compared to the wild-type samples (**Figure 6D**).

### Sexual dimorphic effects on akt/mtor and erk activities in *igf1*-deficient zebrafish

**Figure 7:**
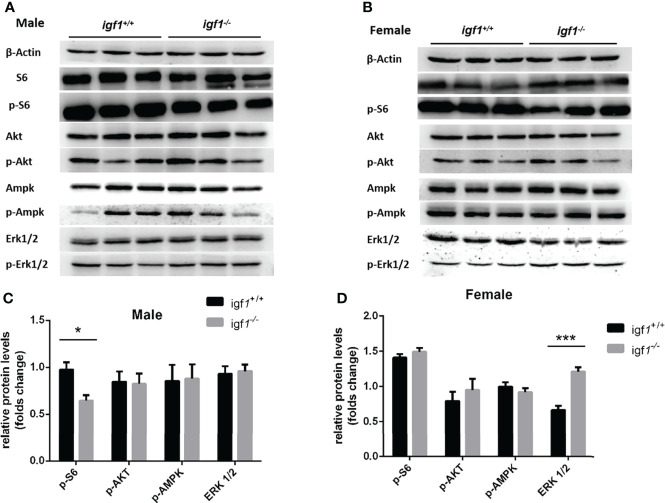
The phosphorylation levels of several key kinases in the hepatic tissue of *igf1*-deficient fish. **(A, B)** Western blot analysis of hepatic actin, p-S6, Akt, p-Akt, AMPK, p-AMPK, Erk1/2, and p-Erk1/2 in *igf1*-deficient male **(A)** and female **(B)** fish. **(C, D)** The data were analyzed by quantifying the protein results using the ImageJ software. Relative expression of p-S6, p-Akt, p-AMPK, and p-Erk1/2 in male **(C)** and female **(D)** zebra fish. *P < 0.05; ***P < 0.001.

Serine/threonine kinase protein kinase B (PKB/AKT), Ribosomal S6 (S6), AMP-activated protein kinase (AMPK), and extracellular signal-regulated kinase (ERK) are key proteins involved in regulation of anabolism and energy metabolism. The phosphorylation status of these proteins normally represent the activities of these kinases (32, 33). Western blotting analysis demonstrated significantly decreased levels of phosphorylated S6K only in mutant males (**Figure 7A, C**), and significantly elevated levels of phosphorylated Erk1/2 only in mutant females compared to their control samples respectively (**Figure 7B, D**).

### Estrogen administration rescues the fatty liver phenotype and AKT-mTOR activity in male *igf1*-deficient zebrafish

**Figure 8:**
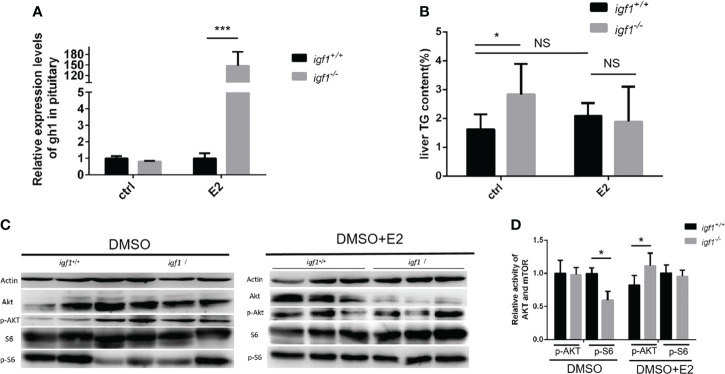
E2 rescue the hepatic steatosis and the AKT-mTOR signaling activity in liver. **(A, B)** Relative expression of gh1 gene in pitutary **(A)** of *igf1*-deficient and wild type treated in E2(100μg/L). Triglyceride composition in liver **(B)** of igf1-deficient and wild type treated in E2(100μg/L). An independent experiment was carried out in which the control group zebrafish was treated with 5% DMSO in fish water. **(C, D)** Western blot analysis of hepatic AKT-mTOR signal activity in male zebrafish. Four animals were sampled for each experiment and three biological repeats were carried out and statistical analysis was performed using a t test (p<0.001, n=3). *p < 0.05, ***P < 0.001.

To define the difference between the male and female in the GH/IGF1 signaling, the adult male zebrafish (90dpf) were exposed to the 17beta-estrodiol (E2) for 14days. The liver tissue of both *igf1* deficient and wildtype zebrafish were collected and checked for the *gh* expression and the TG content. The transcriptional level of *gh* is enhanced with the E2 treatment (**Figure 8A**). No significant difference of the hepatic TG contents between the igf1-deficient males and their controls after E2 treatments (**Figure 8B**). The activity of the AKT-mTOR signaling can be restored after the treatments (**Figures 8C, D**). These results indicate that E2 can rescue, at least partially the defects of fatty liver in igf1-deficient males.

## Discussion

In this study, *igf1*-deficient zebrafish has been generated with CRSPR/Cas9 techniques (**Figure 1**). Instead of severe intrauterine and postnatal growth deficits typically observed in IGF1 deficient mice (3), subtler sexually dimorphic patterns of postnatal growth phenotypes were demonstrated using our *igf1*-deficient fish (**Figure 4**). This implies that the IGF1 function in the GH/IGF1 axis is not essential for survival in zebrafish and the phenotype in somatic growth was moderate. This finding corroborates that of previous reports, which has stated no significant changes of *igf1* expression in *gh* mutant zebrafish during larval stage (14). We have previously observed that insulin signaling can be mediated and/or compensated by the GH/IGF1 signaling in various *insulin receptor a* (*insra*) or *insulin receptor b* (*insrb*)-deficient zebrafish (3). Similarly, it has been observed that significantly elevated expression of *gh1* and *insulin* transcripts exist in our *igf1*-deficient zebrafish (**Figure 2**). This indicates that functional crosstalk between the GH/IGF1 cascade and insulin-like peptides in zebrafish, as well as the elevated levels of *gh1* and *insulin* expression may be due to the compensatory effects. Together, these findings further suggest that IGF1 signaling is not the only essential effector downstream of GH signaling in teleost. Signals other than IGF1, such as Insulin signal, may be important for zebrafish postnatal growth regulation as well.

No sexual dimorphic effects on somatic growth has been reported with classical IGF1 knockout mouse models. This is likely due to severe intrauterine postnatal growth retardation or perinatal lethality caused by overall IGF1 depletion in mice (10). Using a conditional knockout strategy, no differences in body weight in the control and hepatic-specific IGF1 deficient (LID) mice were observed during postnatal growth initially. However, only female LID mice exhibited significantly accelerated growth rates following exogenous recombinant human GH treatment. This indicates the sexual dimorphism in response to GH signaling on growth resulting from defective IGF1 signaling in mice (9). Proceeding with a gene dosage strategy, two partial knockout models of ubiquitous IGF type 1 receptor (*igf1r*) XS mice (strong deficiency mice, with > 50% dosage of floxed *igf1r* deficiency) and M mice (moderate deficiency, ≤ 50% dosage of floxed *igf1r* deficiency) were achieved. Upon comparing the phenotypes of XS and M mice, it has been shown that the significant growth stagnation was twice as severe in XS males as that in XS females. Lipid content per adipocyte was significantly higher in XS males, whereas plasma glucose and insulin levels were lower in XS males. This indicates that the most severe defects observed in these IGF1 receptor deficient models were recorded in XS males (10). These observations also indicate that IGF1 signaling performs in a sexually dimorphic manner in mice.

Enormous literature support that in mammals, AKT/mTOR signaling is the key downstream effective pathway of GH/IGF1 signaling for regulation of anabolic metabolism. S6 is the major downstream target of mTORC1, which stimulates anabolic metabolism (32). AMPK is a major cellular regulator of lipid and glucose metabolism. In order to maintain homeostasis of blood sugar levels, AMPK activation promotes glucose uptake and utilization. MAPK (ERK1/2) is also a key protein involved in regulating anabolic and energy metabolism. The existing crosstalk involving IGF1 signaling and AMPK or MAPK (ERK1/2) activation have been reported in certain types of mammalian tissues (34, 35). The phosphorylation of AKT, S6, AMPK, and MAPK (ERK1/2) generates the activated forms of these kinases in response to their stimulus *in vivo (* 36). In order to test the activation status of these key regulatory signaling molecules in zebrafish hepatic tissue as a result of *igf1*-deficiency, phosphorylated Akt, S6, Ampk and Erk1/2 were analyzed by western blotting. No significant difference in the levels of phosphorylated Ampk was found due to *igf1*-deficiency (**Figure 7**). Significantly decreased levels of phosphorylated S6 observed in *igf1*-deficient males (**Figure 7**) correlated with impaired anabolic metabolism, which is also reflected by the growth retardation seen in the mutant males (**Figure 4**). The significantly elevated activated phosphorylated Erk1/2 levels observed in *igf1*-deficient females (**Figure 7**) may suggest that it actively promotes fatty acid oxidation in the hepatic organ of the female mutants. This is also relevant to features like the low L/P ratios seen in the hepatic tissue of female mutants (**Figure 6**), since it has been suggested that a high hepatic L/P ratio associates with the promoted glycolysis and NADH reductive stress in mouse hepatocytes (37).

Although no drastic growth retardation was observed in the overall sample of mutant fish, a evident sexual dimorphism of zebrafish IGF1 on somatic growth has been observed. Significant growth retardation and fatty liver have been observed in *igf1*-deficient males (**Figures 4**, **5**), but not in females. However, no significant alterations in plasma glucose levels, hepatic *pepck* expression and L/P ratios are seen in igf1-deficient males compared with their controls (**Figure 6**). This might indicate that the elevated levels of insulin expression resulted from the IGF1-deficiency could be sufficient to restore the glucose metabolism in *igf1*-deficient males. However, with even more relative up-regulated levels of *insulin* expression have been seen in the *igf1*-deficient males compared with those of the female mutants (**Figure 2B**), the impaired hepatic Akt/mTORC activation has been seen in *igf1*-deficient zebrafish males, but not in females (**Figures 7A, C** **)**. This might suggest that the insulin signaling might not be major regulator for Akt/mTORC activation in female zebrafish. On the other hand, with the significant up-regulated gh expression resulted from the IGF1 deficiency only seen in the female mutants (**Figure 2A**), together with no altered hepatic Akt/mTORC activation recorded in the liver of *igf1*-decieicent females (**Figures 7B, D** **)**, it might suggest that the GH signaling could be the major mediator responsible for the hepatic Akt/mTORC activation in zebrafish. On other hand, estrogen such as E2 could activate AKT/Tor signaling through bingding GPER which was a G-protein related receptor (18). Insulin expression is augmented significantly in liver of *igf1*-dificient especially male zebrafish. Therefore there was the tendency that the blood glucose level was decreased moderately although with out statistical significant difference. Hypoglycemia was examined in male *igf1*-deficient zebrafish with IGF1 protein administration. It suggested that Igf1 and Insulin could have a superimposed effect in the glucose metabolism in male zebrafish. At the same time, IGF1 protein could rescue the hyperglycemia phenotype in *igf1*-deficient zebrafish (**Figure 6A**).

However, even with the higher levels of *gh* and *insulin* transcripts found in *igf1*-deficient zebrafish females, significant alterations of plasma glucose, hepatic *pepck* expression and L/P ratios were observed in female mutants, but not male mutants (**Figures 5A–C**). Furthermore, significantly reduced levels of 2DG6P were found only in the hepatocytes from *igf1*-deficient females compared to those of their controls (**Figure 5D**), which suggest that relatively more severe defects of the plasma glucose regulation, hepatic gluconeogenesis and cellular glucose uptake occurred in female mutants compared to male mutants. This might suggest the enhanced GH and insulin signals, seen in *igf1*-deficient females, are responsible for maintain the hepatic Akt/mTOR activation caused by the IGF1 deficiency in female (**Figures 6B, D**), but not sufficient to retain the regular glucose metabolism processes caused by IGF1 deficiency in female zebrafish. A high hepatic L/P ratio indicates the promoted glycolysis and NADH reductive stress in mouse hepatocytes (37). This might suggest that the metabolism features of the *igf1*-deficient females are the high hepatic lipid mobilization associated and impaired hepatic glucose uptake and glycolysis (**Figures 4D**, **5**, **6D**). However, a relative normal growth performance of *igf1*-deficient females can be managed to achieve even with the defective glucose metabolism syndrome caused by the loss of IGF1 (**Figure 3**). This might be due to the fact of the high tolerance to hyperglycemia in fish, as many previous reports suggested (38, 39).

It would be always interesting to know whether this sexual dimorphism is mediated by gonadal steroid signals. It has been reported previously that the estrogen could stimulate the GH signaling or even the Akt/mTORC activation (40). The fact of the significant up-regulated *gh* expression is only seen in *igf1*-deficient females, but not in males (**Figure 2A**). Intriguingly, when the *igf1*-deficient males were treated with E2, the up-regulated *gh* expression can be achieved (**Figure 8A**). The hepatic TG contents and phosphor-S6 levels in mutant males can be restored compared with their controls. In addition, the hepatic levels of phosphor-Akt can be up-regulated in igf1-deficient males compared to those of their control fish (**Figures 8**). Therefore, it could be the relatively high estrogen signals presented responsible for the maintenance of the Akt/mTORC activation in the igf1-deficient female. However, the detailed mechanisms of the estrogen action on elevating of *gh* expression or Art/mTORC activation directly should be clarified in the following investigation. Recently investigators found stat 5.1 knockout zebrafish showed reduced gh1 expression. The sexual size dimorphism of the mutant line in adult zebrafish was also eliminated. The exact mechanism of gh1 expression regulation was vital to understand the sexual dimorphism (41).

Taken together, our present studies demonstrated that the IGF1 is not essential for somatic growth in zebrafish. Other IGFs and insulin could partially compensate the loss of the IGF1 in zebrafish. In general, a more severe defective growth performance associated the evident fatty liver exhibited in *igf1*-deficient males compared to the females. The IGF1-deficiency in zebrafish tends to cause the hepatic lipid mobilization defects in males, and glucose metabolism defects in females. The sexual dimorphism defects in *igf1*-deficient fish seems to be mediated, at least partially, by the sexual dimorphic estrogen signals between the genders.

## Data availability statement

The original contributions presented in the study are included in the article/**Supplementary Material**. Further inquiries can be directed to the corresponding author.

## Ethics statement

The animal study was reviewed and approved by Animal ethics committee of Institute of Hydrobiology, Chinese Academy of Sciences.

## Author contributions

NZ and JB conducted most of the experiments for this study. TS and CS provided guidance in experimental operation. GZ and TS provided the *igf1* knockout zebrafish. XJ helped in maintaining the fish. QL and JH performed training and provided insights for this work. QL wrote the paper and prepared the figures. ZY initiated and supervised the research team and edited the paper. All the authors approved the final manuscript.

## Funding

This project was supported by the National Key Research and Development Project (2018YFD0900404); National Natural Science Foundation, China (31530077 to ZY and 31972779 to GZ); and the State Key Laboratory of Freshwater Ecology and Biotechnology (2016FBZ05 to ZY).

## Conflict of interest

TS was employed by China Three Gorges Corporation.

The remaining authors declare that the research was conducted in the absence of any commercial or financial relationships that could be construed as a potential conflict of interest.

## Publisher's note

All claims expressed in this article are solely those of the authors and do not necessarily represent those of their affiliated organizations, or those of the publisher, the editors and the reviewers. Any product that may be evaluated in this article, or claim that may be made by its manufacturer, is not guaranteed or endorsed by the publisher.

## Supplementary material

Supplementary Figure 1 Transcriptional levels of igf1 related genes in skeleton tissues. **(A, B)**Relative expression levels of *igf1ra and igf1rb* in *igf1*-deficient fish and the control wild-type fish and three biological repeats were carried out and statistical analysis was performed using a t test (n=3). **(C–F)** Relative expression levels of *igfbp1a*, *igfbp1b*, *igfbp3* and *igfbp7* in *igf1*-deficient fish and the control wild-type fish and three biological repeats were carried out and statistical analysis was performed using a t test (n=3). There was no difference between the two internal reference gene (*β-actin* and *EF1α*). Four animals were sampled for each experiment and three biological repeats were carried out and statistical analysis was performed using a t test (p<0.001, n=3).**, P < 0.01; ***, P < 0.001.
