# Supplementary figures and images for: Sexual dimorphic effects of igf1 deficiency on metabolism in zebrafish
Source: Front Endocrinol (Lausanne). 2022 Jul 29;13:879962. doi: 10.3389/fendo.2022.879962 (PMC9372914; doi:10.3389/fendo.2022.879962)

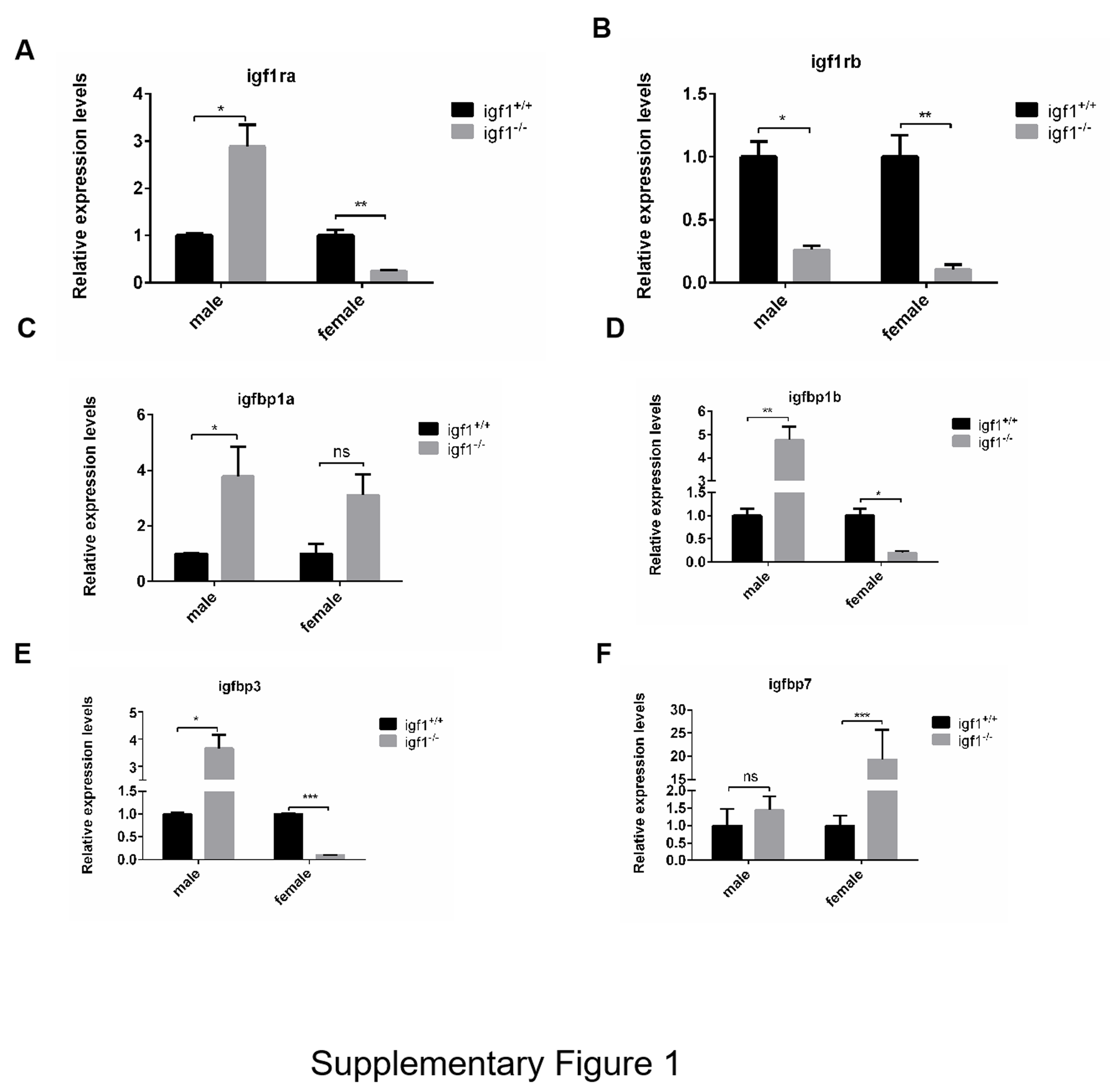

Supplement: Supplementary Figure 1 — Transcriptional levels of igf1 related genes in skeleton tissues. (A, B)Relative expression levels of igf1ra and igf1rb in igf1-deficient fish and the control wild-type fish and three biological repeats were carried out and statistical analysis was performed using a t test (n=3). (C–F) Relative expression levels of igfbp1a, igfbp1b, igfbp3 and igfbp7 in igf1-deficient fish and the control wild-type fish and three biological repeats were carried out and statistical analysis was performed using a t test (n=3). There was no difference between the two internal reference gene (β-actin and EF1α). Four animals were sampled for each experiment and three biological repeats were carried out and statistical analysis was performed using a t test (p<0.001, n=3).**, P < 0.01; ***, P < 0.001. [file Image_1.tif]
